# Triangulating Differential Nonresponse by Race in a Telephone Survey

**Published:** 2007-06-15

**Authors:** Jessica T DeFrank, Bowling J. Michael, Barbara K Rimer, Jennifer M Gierisch, Celette Sugg Skinner

**Affiliations:** Lineberger Comprehensive Cancer Center, The University of North Carolina at Chapel Hill. The author is also affiliated with the School of Public Health, The University of North Carolina at Chapel Hill.; School of Public Health, The University of North Carolina at Chapel Hill, Chapel Hill, NC; School of Public Health, The University of North Carolina at Chapel Hill, Chapel Hill, NC; School of Public Health, The University of North Carolina at Chapel Hill, and the UNC Lineberger Comprehensive Cancer Center, Chapel Hill, NC; Duke Comprehensive Cancer Center, Durham, NC

## Abstract

**Introduction:**

In 1994, the U.S. Department of Health and Human Services mandated sufficient inclusion of racial and ethnic minorities in all federally funded research. This mandate requires researchers to monitor study samples for research participation and differential survey nonresponse. This study illustrates methods to assess differential survey nonresponse when population race data are incomplete, which is often the case when studies are conducted among members of health plans.

**Methods:**

We collected data as part of the PRISM (Personally Relevant Information about Screening Mammography) study, a trial funded by the National Institutes of Health to increase rates of annual mammography adherence. We used two methods to estimate racial distribution of the PRISM study population. The first method, called E-Tech, estimated race of the sample frame by using individuals' names and zip codes. In the second method, we conducted interviews with a subsample of PRISM study refusals. We validated both estimation methods through comparisons with self-reported race. We used race information generated by E-Tech, interviewer estimates, and self-report to assess differential nonresponse in the PRISM study.

**Results:**

The E-Tech method had moderate sensitivity (48%) in estimating race of black participants but higher specificity (97%) and positive predictive value (71%). The interviewer-estimation method had high sensitivity (100%), high specificity (95%), and moderate positive predictive value (80%). Black women were less likely than white women to be reached for study participation.

**Conclusion:**

There was slight differential nonresponse by race in the PRISM study. Techniques described here may be useful for assessing differential nonresponse in samples with incomplete data on race.

## Introduction

Differential nonresponse is a potential problem in all health survey research. It can be particularly problematic in studies that include low-income groups, racial and ethnic minority groups, or both. Differential nonresponse occurs when one sample subgroup has a lower survey response than other subgroups. Statistical strategies to compensate for differential nonresponse, such as weighting, attempt to attenuate the impact of differential nonresponse on survey error (1). However, dissimilarity between participants and nonparticipants on social variables, such as race, sex, education, or income conceal differences on key analysis variables, limiting interpretation and generalizability of study findings (2,3). This phenomenon, known as nonresponse bias, has been identified even in studies with response rates greater than 80% (4,5). Thus, all health survey studies should assess potential nonresponse bias.

Blacks often have lower rates of participation in health survey research compared with whites. This discrepancy is attributed to factors such as socioeconomic status as well as challenges in research recruitment and participation. Studies have found black populations less likely to be located and reached and more likely to refuse participation (6-8). Other barriers to research participation may include general distrust, perceived exploitation in past research studies (e.g., Tuskegee Syphilis Study), doubt about whether participation will result in improved outcomes, and concerns about personal burdens, risks, or costs associated with participation (9-12). Because blacks are often underrepresented in health survey research, the extent to which findings can be generalized to this population is sometimes limited.

In 1994, the U.S. Department of Health and Human Services mandated sufficient inclusion of racial and ethnic minorities in all federally funded research (13). This mandate has prompted researchers to develop enhanced recruitment strategies (14-18) and to closely monitor study samples for potential differential survey nonresponse by race and ethnicity (6). In many instances, researchers can determine whether there is differential survey nonresponse by comparing characteristics of study participants, such as race, to the characteristics of the population or sample frame. Sometimes, however, the data required to make these comparisons are unavailable. Comparison data were unavailable for our PRISM (Personally Relevant Information about Screening Mammography) study, which sampled female members of the North Carolina Teachers' and State Employees' Comprehensive Major Medical Plan, also known as the State Health Plan (SHP). Like many health insurance plans, the SHP does not collect racial or ethnic information on members. Thus, racial composition of both the sample and the frame was unknown.

Our initial estimate was that approximately 23% of participants in the study's baseline telephone interviews would be black, based on the known racial composition of North Carolina state employees (19) and on the representation of black participants in similar research projects (20). However, as we monitored recruitment, we found fewer black participants than expected. Instead of the estimated 23%, black women comprised 11% of study participants. Although part of this discrepancy may have been due to the study's eligibility criteria, which required that women had a recent mammogram, we did not want to rule out the possibility of differential nonresponse.

The primary aim of the research reported here was to determine whether differential nonresponse by race occurred. Because race data were not available on the frame used to select the sample, we tested our primary aim indirectly, using two approaches to estimate race of nonparticipants. We chose two approaches because each single approach has inherent weaknesses. The first approach, called E-Tech, estimated racial composition of the frame using algorithms based on the names and zip codes of individuals. In the second approach, we conducted brief interviews with a subsample of women who refused participation in the PRISM study. A secondary aim was to compare estimated race data with self-reported race data to validate these approaches.

## Methods

### Study sample

PRISM, part of the National Institutes of Health's (NIH's) Health Maintenance Consortium, is an NIH-funded intervention trial to increase rates of mammography maintenance (21). The target population for PRISM is insured women who are adherent to mammography based on national screening guidelines. PRISM identified potential participants through the SHP. The sample frame included North Carolina female residents who were enrolled with the SHP for 2 or more years before sampling, had their last screening mammograms between September 2003 and September 2004 (to ensure that all women had recent, on-schedule mammograms), had only one mammogram within the designated timeframe (to exclude those who had diagnostic mammograms), had no personal history of breast cancer, and were between the ages of 40 and 75. Researchers calculated the target study enrollment as approximately 3545 participants and randomly selected 9079 women from the larger sample frame of 27,944 women for recruitment. The large sample was chosen because we knew from previous studies with similar populations that many women would not meet the described eligibility criteria upon contact.

### Procedures

PRISM study recruitment occurred between October 2004 and April 2005. Researchers first mailed invitation letters to the sample of 9079 potential participants. The letters provided instructions for opting out of the study. In addition, potential participants were sent required HIPAA (Health Insurance Portability and Accountability Act of 1996) information about the types of personal health information that would be collected. Trained telephone interviewers from Battelle Centers for Public Health Research and Evaluation contacted potential participants to obtain their active consent. Following consent, women completed 30-minute baseline telephone interviews designed to collect sociodemographic data (including race) and information on mammography knowledge, beliefs, and practices. Interviewers made up to 12 attempts to contact women. The Institutional Review Boards for the University of North Carolina School of Public Health and Duke University Medical Center approved the research study.

### Participants

PRISM telephone interviewers attempted to contact the random sample of 9079 individuals who met initial eligibility criteria (Figure). Of these, 3543 completed baseline telephone interviews, and 2016 refused participation. Researchers classified 260 women as ineligible upon contact (e.g., too ill, breast cancer history). The remaining women were classified as unknown eligibility because they could not be contacted (n = 838) or because they were removed from the sample (n = 2422) when their enrollment was no longer needed to reach the target sample size. The range in response rates based on the American Association for Public Opinion Research Standard Definitions was 47% to 64% (22). The lower response rate excludes a portion of women with unknown eligibility from the response rate computation; the higher response rate excludes all women with unknown eligibility.

**Figure:**
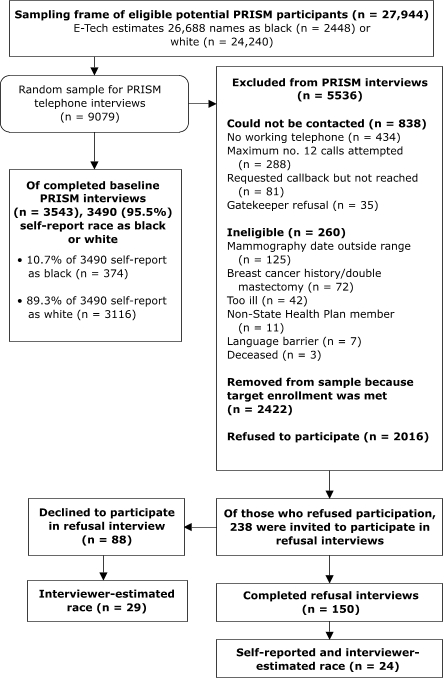
PRISM (Personally Relevant Information about Screening Mammography) participant recruitment for baseline and refusal interviews.

Of the 3490 PRISM study participants for analysis in this study, 89.3% reported their race as white (n = 3116), and 10.7% reported their race as black or African American (n = 374). Fewer than 1% of participants (n = 53) were American Indian or Alaska Native (n = 34), Asian (n = 11), or Native Hawaiian or Other Pacific Islander (n = 1), or gave a response of "other" (n = 7). Twenty-one participants reported that they were Hispanic/Latina.

### Estimating race through Ethnic Technologies

We employed Ethnic Technologies, LLC, a professional data encoding service (23), to estimate race for the entire sample frame of 27,944 women. Ethnic Technologies uses an encoding system, called E-Tech, which inputs subjects' first names, surnames, and geographic locators (ZIP+4 code) into an algorithm to generate race and ethnicity estimates. Ethnic Technologies maintains a database of more than 448,350 unique surnames by ethnicity, 67,695 first names common to more than one ethnicity, and 68,500 first names unique to a given ethnicity. As standard procedure, E-Tech considers the first names of individuals to determine whether those names match ethnically unique first names in its reference files. When there is a match with the first name, the analysis is complete, and a code is set. If there is no match, the surnames of individuals are considered. The algorithm also applies geographic locators to determine, for example, whether an individual resides in a predominately black geographical area but uses a surname common to other racial or ethnic groups. When such a situation occurs, the geographic indicator is used, and the subject is recoded as black. A complete explanation of the E-Tech process is described elsewhere (23). Ours is the first study that used E-Tech information to assess differential nonresponse in a health survey. E-Tech identified a unique race code for 26,979 of the 27,944 women in the entire sample frame (96.5%). The remaining 965 women (3.5%) were coded by E-Tech as unidentified or multiethnic. Of the 27,944 women in the original sample frame, E-Tech estimated 26,688 (95.5%) as either white (n = 24,240) or black (n = 2448).

### Estimating race through refusal interviews 

As a second method to estimate race and to determine whether PRISM study refusals were disproportionately black, we conducted brief interviews, referred to as *refusal interviews*, with a subset of women who were unwilling to participate in the PRISM study (Figure). We conducted the refusal-interview component in March 2005. We solicited participation from 238 women to reach our target of 150 completed refusal interviews (63.0%). These 238 women were not randomly selected; they were instead among the last consecutive potential participants contacted for PRISM study recruitment. The refusal interview contained five items, including a self-reported measure of race as white, black or African American, Asian, Native Hawaiian or Other Pacific Islander, American Indian, Alaska Native, or "other."

Upon completion of 126 of the 150 target refusal interviews, we added a second element to the remaining attempted interviews: we asked interviewers to estimate race of women with whom they spoke, regardless of their participation in refusal interviews, based on verbal cues. The purpose of the interviewer-estimation component was to determine the accuracy of this method through comparisons with self-reported race. PRISM study researchers provided no training to interviewers about how to use verbal cues. Interviewers classified the 53 women with whom they spoke as black or white; none of the women was classified as "do not know" or "other." Of the 53 women contacted by interviewers, 24 agreed to participate in refusal interviews and provided self-reported race. Therefore, we validated the interviewer-estimation method by using a subsample of 24 women.

### Statistical analysis 

We dichotomized race as white and black because the distribution of PRISM study participants was predominately white (89.3%) or black (10.7%). Participants who represented other racial or ethnic groups (<1%) were removed from analyses as were participants who gave a self-reported race as "other" (<1%) because their numbers were too small for meaningful analysis.

**Assessing accuracy of the two methods for estimating race**

We calculated each method's sensitivity, specificity, and positive predictive value to correctly estimate black race compared with self-reported race (24). *Sensitivity* was defined as the probability of correctly estimating black race (true positive). *Specificity* was defined as the probability of correctly estimating white race (true negative). *Positive predictive value* was defined as the probability that an individual was self-reported black (true positive), given an estimation of black (true positive + false positive). We generated κ statistics as additional measures of agreement between self-reported race and estimated race (25).

**Assessing potential differential nonresponse by race**

We used chi-square tests when comparing racial distributions of participants to nonparticipants. We used one-sample binomial tests (z scores) when making comparisons to the PRISM sample frame. Because we found that the E-Tech method tended to misclassify black participants as white, we applied ratio-weighted adjustments to the sample frame. We applied a ratio-weighted adjustment of 1.465 to each black woman identified by E-Tech to increase the proportion of estimated blacks in the frame and applied an adjustment of 0.953 to each estimated white woman to decrease their representation in the frame. We calculated these adjustments through comparisons with self-reported race data. We performed data analyses using SAS version 9.1 (SAS Institute Inc, Cary, NC). Statistical tests were considered significant at *P* < .05; all tests were two-sided.

## Results

### Methods to estimate race

Table 1 shows that the overall level of agreement between the E-Tech estimation of race and self-reported race for PRISM study participants was moderate (κ = 0.53; 95% confidence interval [CI], 0.48–0.58). The positive predictive value for the E-Tech method to estimate black participants was 71.0% (174/245). The probability of a black participant being correctly estimated by E-Tech as black (sensitivity) was 47.6% (174/365). The probability of a white study participant being correctly estimated by E-Tech as white (specificity) was 96.9% (2916/3010). E-Tech misclassified 190 black participants (52% of all black participants) as white, whereas 71 white participants (2% of all white participants) were misclassified as black. The data show that the E-Tech method underestimated black race.

We found a high level of agreement between interviewer estimates of race and self-reported race (κ = 0.86; 95% CI, 0.60–1.00) (Table 2). The positive predictive value for the interviewer method to estimate black participants was 80.0% (4/5). All four women who self-reported as black were correctly identified by interviewers as black (100% sensitivity). Nineteen of 20 women who self-reported as white (95.0% specificity) were correctly identified by interviewers as white. One woman who self-reported her race as white was incorrectly classified as black.

### Differential nonresponse by race

Table 3 shows that the self-reported racial distribution of PRISM study participants differed significantly from the weighted and unweighted E-Tech–estimated racial distribution for the sample frame. Table 3 also shows that racial distributions for the weighted and unweighted E-Tech sample frame significantly differed from each other; the weighted adjustments to the E-Tech–estimated sample frame compensated for the E-Tech underenumeration of black women.

Table 4 shows categories of nonparticipation using E-Tech estimates. Compared with the weighted sample frame, women who could not be contacted for participation were disproportionately black. Women who were ineligible for the study, removed from the sample, or refused participation were not disproportionately black compared with the weighted sample frame.

Comparison of refusal-interview participants with the weighted E-Tech sample frame showed slight nonsignificant differences in the percentage of black individuals (Table 5). Comparison of refusal-interview participants with PRISM participants showed that racial distributions were significantly different. The racial distribution of refusal-interview participants did not differ from the estimated racial distribution of women who declined participation in refusal interviews.

## Discussion

### Methods to estimate race

Although E-Tech was nearly perfect in estimating white race when participants were self-reported white, it misclassified 52% of the sample's self-reported black participants as white, resulting in underestimation of black participants. This discrepancy might be explained by the E-Tech process for assigning race codes. For example, if a woman resided in a predominately white geographical area, she was coded as white unless her first or surname suggested otherwise. Black participants whose first or surnames were not ethnically unique (e.g., Melissa Smith) and lived in predominately white geographical areas were likely coded as white. Similarly, a study by Kwok and Yankaskas (26), which used census-block group data to estimate race of women enrolled in a mammography registry, found that black women were accurately identified less consistently than white women. Further investigation is needed to explore how the E-Tech system of identifying black individuals, as well as those methods described by Kwok and Yankaskas and others (27), can be improved. This investigation is critical if researchers are to monitor study samples for potential differential nonresponse when race information is incomplete.

The method in which interviewers estimated race of women with whom they spoke was highly accurate. Only one white study participant was misclassified as black; the rest were accurately identified. This finding is consistent with literature suggesting that certain characteristics of African American vernacular English may make it distinguishable from non-African American speakers (28). Although the interviewer-estimation method appears to be preferable because interviewers almost always judged race of a woman correctly, the sample size for this supplemental validation experiment was small (n = 24) and should be considered exploratory. In addition, we cannot know for certain how much the outcome was influenced by the fact that most interviewers were black women. Future studies should attempt to replicate this finding with larger samples. Also, further research is needed to determine whether estimating race through spoken language can be extended to men, to other age groups and geographical regions, and to racial or ethnic backgrounds other than black. Interviewers for this study did not receive training on using verbal cues to estimate race. It is likely that the accuracy of this estimation method could be improved through formal training.

### Assessing differential nonresponse by race

By triangulating results from multiple statistical comparisons, we assessed potential differential nonresponse in the PRISM study. Both unweighted and weighted E-Tech–estimated sample frames differed in their racial distributions compared with PRISM study participants, leading us to conclude there was slight differential nonresponse by race.

When we examined categories of nonparticipation using weighted E-Tech estimates, we found that study interviewers had more difficulty reaching black women compared with white women. That is, the category of nonparticipants who had no working telephones, reached the maximum number of call attempts without successful contact, requested call-backs but were not reached on subsequent attempts, or for whom gatekeepers refused participation was disproportionately black. The finding that black women were more difficult to reach is consistent with reports in the health survey literature (7,8). Enhanced recruitment methods and strategies are needed to ensure that federal research achieves appropriate participation of racial and ethnic populations (14).

Our findings were inconclusive as to whether study refusals were disproportionately black. Although racial distribution of refusal-interview participants was slightly different compared with the distribution of the weighted E-Tech sample frame, the differences were not statistically significant. Yet, racial distribution of refusal-interview participants was significantly different compared with PRISM study participants. Findings in the health-survey literature suggest that blacks may be more likely to refuse participation. For example, analysis of the 2003 Behavioral Risk Factor Surveillance System (BRFSS) data found refusal rates were significantly higher in counties with higher percentages of black residents (6). However, the fact that our study sample was composed of women who were adherent to mammography guidelines at entry may explain why our findings may have differed from studies examining more general samples.

### Limitations

The two described methods to estimate race of the study sample each had limitations. First, the E-Tech method tended to misclassify black women as white. We applied weighted adjustments to the E-Tech numbers to help overcome this limitation. Second, because we implemented the refusal-interview component as a supplemental study near the latter stages of participant recruitment, sample sizes used to assess the accuracy of the interviewer-estimation method were small and should be replicated with larger samples. Given the characteristics of our PRISM sample, our findings are limited in their generalizability. For example, we do not know the accuracy of the E-Tech and interviewer methods to estimate race for men or age groups such as adolescents. Also, our sample had very few participants who represented racial or ethnic groups other than black and white. Thus, we do not know how accurate these methods would be for estimating race or ethnicity for Hispanics, Asians, or other groups. Our findings as they relate to differential nonresponse are generalizable only to our target population of insured women who are adherent to mammography.

### Conclusion

Adequate participation in health research from racial and ethnic minorities is essential to reveal potential health disparities, to ensure that results of intervention and other research can be generalized to these populations, and to comply with federal regulations. Monitoring recruitment is essential to determine whether study participants are disproportionate in their racial composition compared with the sample and, furthermore, whether conclusions drawn from study findings may be limited in their generalizability due to nonresponse bias. We illustrated two methods to assess differential nonresponse when race data are incomplete. Like many studies that rely on samples from government or health plan populations, racial or ethnic data were not available for our initial sample. Techniques described here may be useful to other researchers who wish to assess potential differential nonresponse when faced with incomplete race data. Use and improvement of methods, such as E-Tech and telephone-interviewer estimation of race, are important given U.S. Department of Health and Human Services requirements to achieve adequate participation from racial and ethnic minority groups in federally funded health research (13). With declining survey response rates in the United States (29), it is critical that we understand whether survey research is underrepresenting some population groups. One of the potential values of the methods described here is that they can be used in real time to determine whether there are imbalances in research participation. If an imbalance is found, corrective action could be taken while studies are underway.

## Figures and Tables

**Table 1:** Agreement Between E-Tech Estimate of Race and Self-reported Race for PRISM Study Particpants (n = 3375), North Carolina, 2005^a^

Category	Black	White	American Indian,Asian,or Native Hawaiian	Total
Self-reported race	365	3010	0	3375
E-Tech–estimated race	245	3106	24	3375
Correctly identified	174	2916	0	3090
Incorrectly identified^b^	71	190	24	285

PRISM indicates Personally Relevant Information About Screening Mammography.

a E-Tech did not identify race codes for 115 of the 3490 PRISM study participants who self-reported black or white, resulting in 3375 total participants. Sensitivity of E-Tech estimates of black participants = 47.7% (174/365); positive predictive value of E-Tech estimates of black participants = 71.0% (174/245); specificity of E-Tech estimates of white participants = 96.9% (2916/3010). κ = 0.53; 95% confidence interval, 0.48–0.58.

b Of the 71 participants who were incorrectly identified as black, all self-reported as white. Of the 190 participants who were incorrectly identified as white, all self-reported as black. Of the 24 participants incorrectly identified as American Indian, Asian, or Native Hawaiian, 1 self-reported as black and 23 self-reported as white.

**Table 2:** Agreement Between Telephone-Interviewer Estimate of Race and Self-reported Race Among Subsample (n = 24) of Refusal-Interview Participants, North Carolina, 2005^a^

Category	Black	White	Total
Self-reported race	4	20	24
Interviewer-estimated race	5	19	24
Correctly identified	4	19	23
Incorrectly identified	1	0	1

PRISM indicates Personally Relevant Information About Screening Mammography.

a Sensitivity of interviewer estimates of black participants = 100% (4/4); positive predictive value of interviewer estimates of black participants = 80% (4/5); specificity of interviewer estimates of white participants = 95% (19/20). κ = 0.86; 95% confidence interval, 0.60–1.00.

**Table 3:** Self-Reported Race for PRISM Study Participants Compared With Unweighted and Weighted E-Tech–Estimated Sample Frames, North Carolina, 2005

Race	PRISM Participants (Self-Reported Race) (n = 3490) % (95% CI)	Unweighted PRISM Frame (E-Tech-Estimated Race)^a^ (N = 26,688) % (95% CI)	Weighted PRISM Frame (Adjusted E-Tech-Estimated Race)^b^ (N = 26,688) % (95% CI)
Black	10.7 (9.7-11.7)	9.2 (8.8-9.5)	13.4 (13.0-13.8)
White	89.3 (88.3-90.3)	90.8 (90.4-91.2)	86.6 (86.2-87.0)

PRISM indicates Personally Relevant Information About Screening Mammography; CI, confidence interval. N values are actual (unweighted) values whereas proportions are weighted proportions.

a PRISM participants compared with E-Tech–estimated PRISM frame: *z* = 3.10, *P* = .002.

b PRISM participants compared with weighted E-Tech–estimated PRISM frame: *z* = −4.65, *P* < .001. Unweighted E-Tech–estimated PRISM frame compared with weighted E-Tech–estimated frame: *z* = 23.97, *p* < .001.

**Table 4:** Weighted E-Tech–Estimated Racial Distributions for Categories of Nonparticipation Compared With Weighted PRISM Frame, North Carolina, 2005

Race	Weighted PRISM Frame (Adjusted E-Tech- Estimated Race) (N =26,688)% (95% CI)	Weighted E-Tech-Estimated Nonparticipants			

		Could Not Be Contacted^a^ (n = 796)% (95% CI)	Ineligible for Study^b^ (n = 246)% (95% CI)	Removed From Sample^c^ (n = 2324)% (95% CI)	Refused^d^ (n = 1942) % (95% CI)
Black	13.4 (13.0-13.8)	18.3 (15.6-20.9)	10.7 (6.8-14.6)	14.0 (12.6-15.4)	13.5 (12.0-15.0)
White	86.6 (86.2-87.0)	81.7 (79.1-84.3)	89.3 (85.4-93.2)	86.0 (84.6-87.4)	86.5 (85.0-88.0)

PRISM indicates Personally Relevant Information About Screening Mammography; CI, confidence interval. N values are actual (unweighted) values whereas proportions are weighted proportions.

a Comparison with weighted PRISM frame: *z* = 4.07, *P* < .001.

b Comparison with weighted PRISM frame: *z* = −1.24, *P* = .22.

c Comparison with weighted PRISM frame: *z* = 0.91, *P* = .36.

d Comparison with weighted PRISM frame: *z* = 0.12, *P* = .91.

**Table 5:** Analysis of Refusal-Interview Participants, PRISM Study, North Carolina, 2005

Race	Refusal-Interview Participants (Self-Reported Race) (n = 150)% (95% CI)	Weighted PRISM Frame (Adjusted E-Tech Estimated Race)^a^ (N = 26,688)% (95% CI)	PRISM Participants (Self-Reported Race)^b^ (n = 3490)% (95% CI)	Declined Refusal Interview, (Interviewer-Estimated Race)^c^ (n = 29)% (95% CI)
Black	16.7 (10.7-22.6)	13.4 (13.0-13.8)	10.7 (9.7-11.7)	17.2 (3.5-31.0)
White	83.3 (77.4-89.3)	86.6 (86.2-87.0)	89.3 (88.3-90.3)	82.8 (69.0-96.5)

PRISM indicates Personally Relevant Information About Screening Mammography; CI, confidence interval. N values are actual (unweighted) values whereas proportions are weighted proportions.

a Refusal-interview participants compared with weighted PRISM frame: *z* = 1.17, *P* = .24.

b Refusal-interview participants compared with PRISM participants: χ^2^ = 5.2;* P* = .02.

c Refusal-interview participants compared with those who declined refusal interview and for whom interviewer estimated race: χ^2^ = 0.06;* P* = .94.
